# The speciation history of northern‐ and southern‐sourced *Eranthis* (Ranunculaceae) species on the Korean peninsula and surrounding areas

**DOI:** 10.1002/ece3.4969

**Published:** 2019-02-14

**Authors:** Ami Oh, Byoung‐Un Oh

**Affiliations:** ^1^ Department of Biology Chungbuk National University Cheongju Chungbuk Korea

**Keywords:** ancestral area reconstruction, *Eranthis*, genetic diversity, genetic structure, origin, phylogeny, phylogeography, speciation, the Korean peninsula

## Abstract

The temporal and spatial origins and evolution of the genus *Eranthis* have not been previously studied. We investigated the speciation and establishment histories of four *Eranthis* species: *Eranthis byunsanensis*, *E. pungdoensis*, *E. stellata*, and *E. pinnatifida*. The sampling localities were Korea, Japan, Jilin in China, and the area near Vladivostok in Primorskiy, Russia. We used 12 chloroplast microsatellite loci (*n* = 935 individuals) and two chloroplast noncoding regions (*rpl16* intron, *petL*‐*psbE* intergenic spacer; *n* = 33 individuals). The genetic diversity, genetic structure, phylogenetic relationships of the four species were analyzed, and their ancestral areas were reconstructed. The high genetic diversity of the Jeju island population of *E. byunsanensis* and Russian populations of *E. stellata* indicated these species’ northward and southward dispersal, respectively. The genetic structure analyses suggest that the populations in these four species have limited geographical structure, except for the Chinese *E. stellata* population (SCP). The phylogenetic analyses suggest that *E. byunsanensis* and *E. pinnatifida* are sister species and that Chinese SCP may not belong to *E. stellata*. The ancestral area reconstruction revealed that the most recent common ancestor of the four species existed in the current Chinese habitat of *E. stellata*. This study shows that *E. byunsanensis* and *E. pinnatifida* originated from a southern *Eranthis* species and speciated into their current forms near Jeju island and near western regions of Japan, respectively, during the Miocene. *E. stellata* may have dispersed southward on and near the Korean peninsula, though its specific origin remains unclear. Interestingly, the Chinese *E. stellata* population SCP suggests that the Chinese population might be most ancient among all the four *Eranthis* species. *E. pungdoensis* may have allopatrically speciated from *E. byunsanensis* during the Holocene. The Korean peninsula and the surrounding areas can be considered interesting regions which provide the opportunity to observe both northern‐ and southern‐sourced *Eranthis* species.

## 1 INTRODUCTION

The Korean peninsula and surrounding areas, including Japanese archipelago, East Sea, Yellow Sea, East China Sea, and the Chinese and Russian regions near North Korea, have a variety of geographical features and have experienced a number of dynamic orogenic and ecological events. This has led to diverse distributional patterns for a wide range of species in these regions. For example, Korea, which is surrounded on three sides by sea and contains large mountainous regions, exhibits high geographical complexity and provides the combinations of diverse geography and ecology for many taxa on this area. Also, interestingly, these regions can be appropriate for observing many closely related species exhibiting various distributions.

To aid in our understanding of various distributional patterns of the taxa in these regions and investigate their origins and speciation histories based on these patterns, we propose the genus *Eranthis* (Ranunculaceae) as an intriguing model for evolutionary study. The genus *Eranthis* is an early flowering herbaceous plant which has a tuberous rhizome, petaloid sepals, and palmately divided leaves. This genus is made up of 8–9 species that range from Europe to East Asia, and exhibits interesting distributions. This genus generally exhibits high levels of endemism and is distributed in both mainlands and islands. Its constituent species seldom co‐occur, and the sizes of these species’ distributional ranges usually vary much.

**Figure 1:**
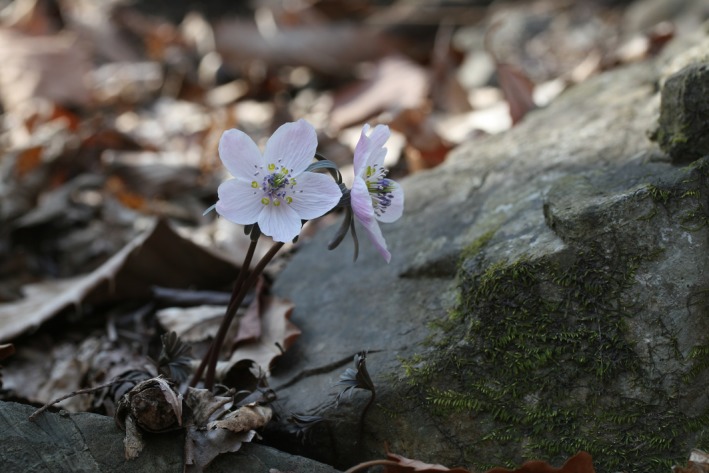
*Eranthis byunsanensis* of Kyoungju, Republic of Korea

A number of morphological, ecological, and genetic studies have been conducted on the genus *Eranthis*. For example, previous research has investigated the genetic variation of the populations of the Korean endemic *Eranthis byunsanensis* (Figure 1) and the environmental characteristics of its habitat (Kim et al., 2012; So, Lee, & Park, 2012). *E. byunsanensis* and its closely related species *Eranthis pungdoensis* were compared based on their genetic variation, and taxonomic analysis was conducted for these two species (Lee, Yeau, & Lee, 2012). In addition, the seeds of *E. byunsanensis* and *E. stellata* have been compared morphologically (Jung, Shin, & Heo, 2010), the flowering rate and pollen release of *E. hyemalis*, which inhabits Europe (Rysiak & Żuraw, 2011), have been analyzed, and cytological studies on *E. stellata* and *E. pinnatifida* have been conducted (Kurita, 1955; Yuan & Yang, 2006). Despite this past research interest, however, the origin and evolution of this genus have not received much attention. In this study, we aimed to investigate the speciation as establishment histories of this genus on the Korean peninsula and in nearby regions, thereby adding to the basic knowledges of this genus, and gaining important evolutionary insights into its constituent species.

*Eranthis* species are morphologically clustered into two groups: one with yellow sepals ranging from Western Europe to Central Asia, and the other with white sepals ranging from Central China to Russia, Korea, and Japan. Here, we focus on four *Eranthis* species with white sepals: (a) *Eranthis stellata*, found all across the Korean peninsula, in Jilin and Liaoning in China, in the western land near the Sea of Okhotsk and in Primorskiy Krai, in eastern‐most Russia; (b) *Eranthis pinnatifida*, endemic to Japan and found in the central and southern areas of Honshu; (c) *E. byunsanensis*, endemic to Korea and found across the Korean peninsula, with its northern limit possibly in North Korea; and (d) *E. pungdoensis*, endemic to Korea and found only on the small island of Pungdo in the Yellow Sea (Oh & Ji, 2009; Oh et al., 2016; Sun, Kim, & Kim, 1993). We analyzed 12 chloroplast microsatellites and two chloroplast noncoding regions in these species, looking specifically at (a) the genetic diversity of the populations of each species; (b) the genetic structure of each species; (c) phylogenetic reconstructions at both population and species levels; (d) the divergence times for each lineage and species; and (e) ancestral area reconstructions.

In this study, we examined to which direction these four *Eranthis* species dispersed in the past, and where and when they speciated. We revealed that the Korean peninsula, as a contact area for “northern” *E. stellata* and “southern” *E. byunsanensis* and *E. pungdoensis*, can offer the opportunity to infer the origins of these species, and that the origin of Japanese *E. pinnatifida* can also be analyzed in relation to other *Eranthis* species in Korea. In short, our research helps to clarify the current and historical distributions of these species, to determine their origins and speciation patterns, and at the same time, to reinforce the utility of the Korean peninsula and its neighboring areas as subjects of evolutionary biology.

## 2 MATERIALS AND METHODS

### 2.1 Plant materials and sampling

**Figure 2:**
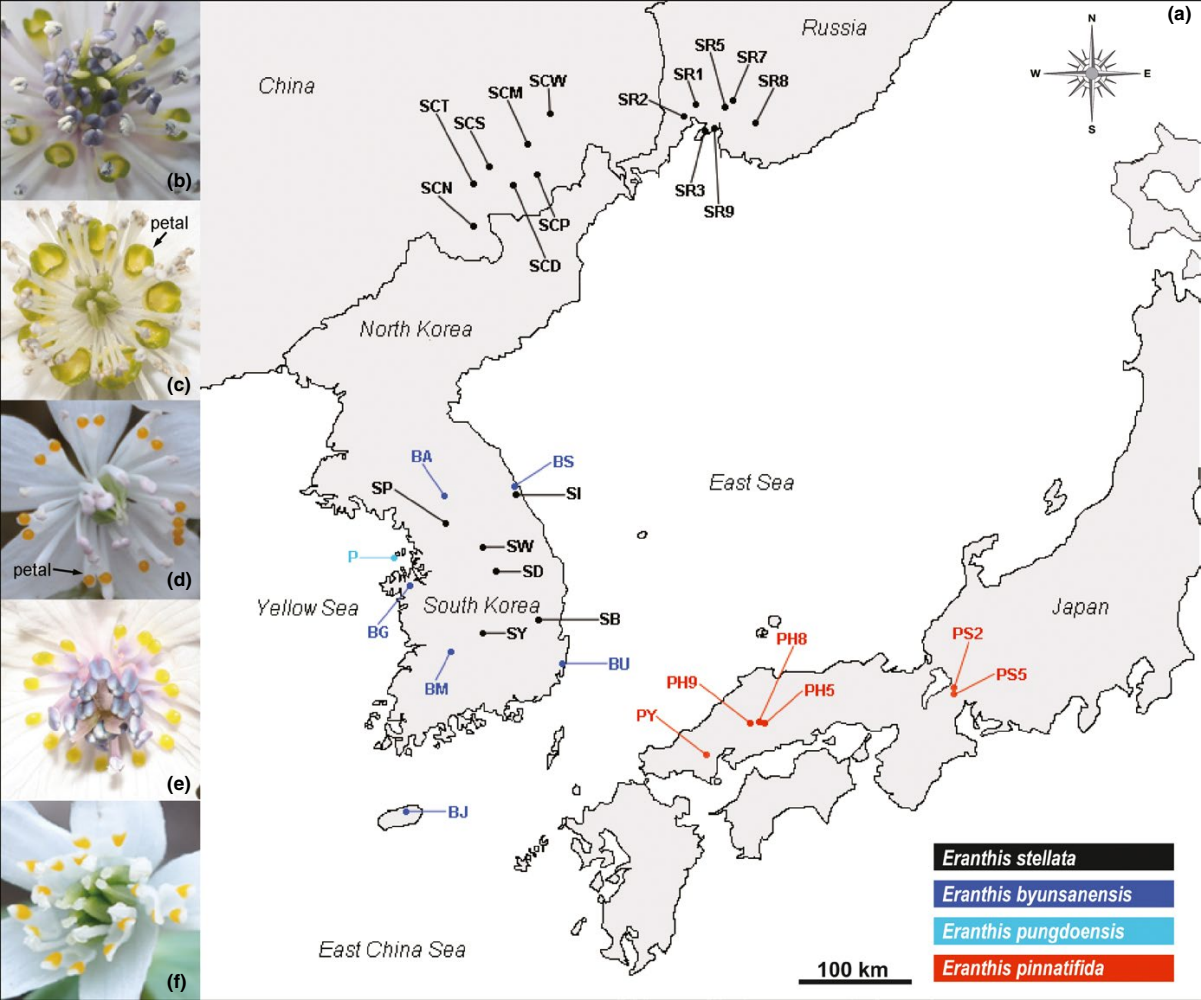
(a) Collection sites of four *Eranthis* species, *E. stellata*, *E. byunsanensis*, *E. pungdoensis*, and *E. pinnatifida*. Black dots denote the material sampling sites for *E. stellata*; blue dots for *E. byunsanensis*, light blue dot for *E. pungdoensis*; and red dots for *E. pinnatifida*. (b) Image of pistils, stamens, and petals of *E. byunsanensis*, (c) *E. pungdoensis*, (d) *E. stellata*, (e) *E. pinnatifida*, and (f) *E. albiflora*

We collected bracts or leaves from 935 individuals in four species of the genus *Eranthis*, with up to 30 individuals sampled for each population, that is, 572 individuals from 20 populations for *E. stellata*, 162 individuals from six populations for *E. byunsanensis*, 30 individuals from one population for *E. pungdoensis*, and 171 individuals from six populations for *E. pinnatifida* (Figure 2a; Supporting information Table S1 in Appendix S1). The sampled individuals were about 1 m apart from each other. For *E. stellata*, the sampling sites do not represent its entire distributional ranges, with the North Korean and the majority of the Russian populations not sampled. Since *E. byunsanensis* and *E. pungdoensis* are morphologically almost same except for the size of their petals (Figure 2b,c), and it is inferred that *E. pungdoensis* diverged from *E. byunsanensis* not very long ago, they were analyzed together as if they were the same species.

*Eranthis albiflora*, *E. longistipitata*, *E. hyemalis*, *Actaea asiatica,* and *Cimicifuga simplex* were also sampled as outgroups.

The sampled bracts or leaves were dried with silica gel, and the total genomic DNA was extracted using DNeasy Plant Kit (QIAGEN, Seoul, Korea). The voucher specimens were deposited in the herbarium of Chungbuk National University.

### 2.2 cpSSR genotyping and cpDNA sequencing

All of the 935 individuals of the four *Eranthis* species were genotyped at 12 chloroplast microsatellite loci (Ebp01, Ebp40, Ebp27, Ebp31, Ebp25, Ebp12, Ebp10, Ebp06, Ebp38, Ebp11, Ebp28, Ebp32) which were randomly selected from the 24 cpSSR loci isolated by Oh and Oh (2017). The PCR procedure was the same as that of Oh and Oh (2017), and the length of PCR products was measured using the ABI3730xl DNA Analyzer (Applied Biosystems) and GeneMapper v. 3.7 (Applied Biosystems).

In addition, one individual from each population of the four focal *Eranthis* species (giving a total of 33 individuals) and five outgroup species, were sequenced at two chloroplast noncoding regions, *rpl16* intron and *petL*‐*psbE* (Shaw et al., 2005; Shaw, Lickey, Schilling, & Small, 2007). Sequencing was conducted in both directions with ABI3730xl DNA Analyzer (Applied Biosystems), and the consensus sequences were created with Sequencher 4.8 (Gene Codes Corp., Ann Arbor, MI, USA).

### 2.3 Data analysis

#### 2.3.1 Genetic diversity

The 12 chloroplast microsatellite markers were used to estimate the genetic diversity of each sampled population. We estimated the number of haplotypes in each population and number of private haplotypes. We also calculated the effective number of haplotypes, haplotype richness, and Nei's index of genetic diversity estimated without bias. These analyses were performed in Haplotype Analysis ver. 1.05 (Eliades & Eliades, 2009).

#### 2.3.2 Population structure

To determine the population structure based on cpSSR loci for the four *Eranthis* species, STRUCTURE 2.3.4 (Pritchard, Stephens, & Donnelly, 2000) was used. Ten independent runs were performed for each *K* (*K* = 1~10), with each run composed of 100,000 burn‐in steps, followed by 100,000 MCMC steps. The admixture model and the correlated allele frequencies model were applied. The optimal number of populations (*K*) was determined using Evanno, Regnaut, and Goudet (2005) with the software STRUCTURE HARVESTER 0.6.94 (Earl & vonHoldt, 2012).

For each species, a spatial analysis of molecular variance (SAMOVA) was performed using the cpSSR data to define groups of populations that are geographically homogeneous and maximally differentiated with each other (SAMOVA 2.0; Dupanloup, Schneider, & Excoffier, 2002). In this analysis, a simulated annealing procedure was used, and the number of initial configuration of groups was set to 100.

The genetic variation among groups of populations, among populations within group, and within populations was estimated using analysis of molecular variance, AMOVA (Excoffier, Smouse, & Quattro, 1992) with Arlequin 3.5 (Excoffier & Lischer, 2010). In this analysis, the groups of populations were defined according to the results of SAMOVA analysis.

The software POPART (Leigh & Bryant, 2015) was used to construct the network of the sequences for the four species, with one sequence from each population, and here, median‐joining method (Bandelt, Forster, & Röhl, 1999) was applied. In the analysis process, the columns with gaps or ambiguous characters in the alignment input files were masked, which often led to originally different sequences becoming identical. Because of this, even though all the sequences were different from one another in the input data, there were haplotypes with more than one sample in the result.

#### 2.3.3 Phylogenetic inferences and divergence time estimation

Bayesian inference and maximum likelihood were applied in the reconstructions of the phylogenetic trees. The two chloroplast noncoding sequences, *rpl16* intron and *petL*‐*psbE*, were aligned with MUSCLE (Edgar, 2004) using the software MEGA7 (Kumar, Stecher, & Tamura, 2016), and manually edited with the same software. These two regions were concatenated (2,293 bp) for the Bayesian analysis of phylogenetic relationships, which was conducted in BEAST 1.8.0 (Drummond & Rambaut, 2007; Drummond, Suchard, Xie, & Rambaut, 2012). One sequence from each population across the four species (33 sequences in total), were analyzed, with *C. simplex *(CSD), *A. asiatica *(ND), *E. longistipitata *(LC), *E. albiflora* (HR) used as outgroups. *E. hyemalis *(HYE), which is European *Eranthis* species, was excluded from this analysis, because the sequencing for *rpl16* intron region in this species was not successful.

In Bayesian inferences, in which tree topology and divergence times were sought, nucleotide substitution model of HKY + G + I and uncorrelated lognormal relaxed clock were applied, and the Yule process was chosen as a tree prior. The length of the MCMC chain was set to 800,000 generations, and parameters were logged every 200 generations. In TreeAnnotator 1.8.0 (Rambaut & Drummond, 2013), burn‐in was set to 400 trees and maximum clade credibility tree was generated with mean height as node height. Software FigTree 1.4.3 (Rambaut, 2017) was used to graphically present the phylogenetic trees.

Since we did not have any fossil record which can be used to estimate the divergence times, we obtained two calibration points from a previous phylogeographical study which included the genus *Eranthis* in its phylogeny (Wang et al., 2016). In that study, the crown group age of the genus *Eranthis*, when including seven extant species in its phylogenetic analysis, was <47 Ma. Also, in another phylogeny reconstruction in the same paper, the most recent common ancestor (MRCA) for *E. stellata*, *A. asiatica,* and *C. simplex* was determined to be 57.55 Ma with 95% highest posterior density of 56.41–59.21.

Maximum likelihood phylogenetic trees were constructed in RAxML 7.0.3 (Stamatakis, 2006) with GTR model. The *rpl16* intron region and *petL*‐*psbE* region were analyzed separately; for the *petL*‐*psbE* region, *E. hyemalis* was included in the outgroup, because the sequencing was successful.

#### 2.3.4 Biogeographical analysis

We performed a statistical dispersal vicariance analysis (S‐DIVA) in the software RASP 4.0 (Yu, Harris, Blair, & He, 2015). In this ancestral area reconstruction, the distributional ranges of the four *Eranthis* species were assigned to four areas: (A) South Korea (*E. byunsanensis*, *E. stellata*), including a small island Pungdo in the Yellow Sea (*E. pungdoensis*); (B) Jilin in China (*E. stellata*); (C) the area around Vladivostok in Primorskiy, Russia (*E. stellata*); and (D) Japan (*E. pinnatifida*).

## 3 RESULTS

### 3.1 Genetic diversity

The genetic variation of the 33 populations from *E. byunsanensis*, *E. pungdoensis*, *E. stellata,* and *E. pinnatifida* was analyzed at 12 chloroplast microsatellites loci (Table 1). For *E. stellata*, the Russian populations were more genetically diverse (H_e; Nei's genetic diversity estimated without bias = 0.680–0.869) than the populations on the Korean peninsula (H_e = 0.439–0.876) and the Jilin region of China (H_e = 0.286–0.821). For *E. byunsanensis*, the southernmost Jeju island population (BJ) was more genetically diverse (H_e = 0.837) than the populations on the Korean peninsula (H_e = 0.353–0.739). For *E. pinnatifida*, in general, the Hiroshima populations tended to exhibit higher genetic diversities (H_e = 0.572–0.674) than other populations (H_e = 0.254–0.709). For *E. pungdoensis*, its only population (H_e = 0.582) seems to need other approaches to be clearly described in the perspective of genetic diversity.

**Table 1:** Genetic diversity estimates for the 33 populations in four *Eranthis* species based on 12 chloroplast microsatellites

Taxon/population	*N*	A	P	N_e	R_h	H_e
*E. stellata*						
SR1	30	8	7	6.250	6.324	0.869
SR2	30	7	6	5.000	5.212	0.828
SR3	30	6	6	2.922	3.888	0.680
SR5	30	10	10	5.556	7.225	0.848
SR7	30	10	9	5.921	7.381	0.860
SR8	29	9	8	4.918	6.849	0.825
SR9	30	7	7	2.961	4.407	0.685
SCW	30	3	3	1.867	1.985	0.480
SCN	30	4	2	2.432	2.507	0.609
SCM	19	4	2	2.798	3.000	0.678
SCS	20	6	2	3.448	4.900	0.747
SCT	26	4	1	1.380	2.397	0.286
SCP	28	5	5	2.085	3.036	0.540
SCD	30	8	8	4.839	6.044	0.821
SY	30	3	3	1.737	1.956	0.439
SP	30	3	3	2.711	1.999	0.653
SD	30	4	4	3.061	2.870	0.697
SI	30	10	10	6.522	7.474	0.876
SW	30	3	3	2.711	1.999	0.653
SB	30	2	2	1.867	1.000	0.480
Mean	28.60	5.80	5.05	3.55	4.12	0.68
*E. byunsanensis, E. pungdoensis*						
BA	30	2	2	2.000	1.000	0.517
BG	25	3	3	1.513	1.941	0.353
BJ	24	9	9	5.053	7.058	0.837
BM	30	6	6	2.647	4.099	0.644
BS	29	2	2	1.578	1.000	0.379
BU	24	4	4	3.429	2.999	0.739
P	30	4	4	2.284	2.507	0.582
Mean	27.43	4.29	4.29	2.64	2.94	0.58
*E. pinnatifida*						
PH5	30	4	4	2.866	2.633	0.674
PH8	30	4	2	2.239	2.267	0.572
PH9	30	3	1	2.663	1.997	0.646
PS2	29	4	4	3.174	2.655	0.709
PS5	23	3	3	1.570	1.974	0.379
PY	29	3	3	1.324	1.622	0.254
Mean	28.50	3.50	2.83	2.31	2.19	0.54

Note

A: number of haplotypes; H_e: Nei's index of genetic diversity estimated without bias; *N*: number of individuals; N_e: effective number of haplotypes; P: number of private haplotype; R_h: haplotype richness.

### 3.2 Genetic structure

**Figure 3:**
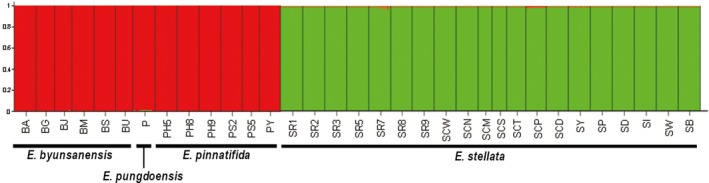
Histogram of genetic assignment analysis with STRUCTURE for all the populations of four *Eranthis* species, at *K* = 2. The population names are listed below the histogram

When the four species were analyzed using STRUCTURE, *E. byunsanensis*, *E. pungdoensis*, and *E. pinnatifida* were mostly assigned to cluster 1, and *E. stellata* to cluster 2. This shows that *E. byunsanensis*, *E. pungdoensis*, and *E. pinnatifida* are genetically closely related, while *E. stellata* does not have a significant relationship with the other three species (Figure 3). STRUCTURE analysis for each species individually was also conducted. In the analysis for *E. byunsanensis* and *E. pungdoensis*, four populations of *E. byunsanensis* (BJ, BU, BM, BA) were assigned to cluster 1, and two *E. byunsanensis* populations (BG, BS) and a population of *E. pungdoensis* (P) were mostly assigned to cluster 2 (Supporting information Figure S1 in Appendix S1). For *E. pinnatifida*, the Hiroshima populations PH5, PH8, PH9 were assigned to cluster 1, the Shiga populations PS2, PS5 assigned to cluster 2, and the Yamaguchi population PY to cluster 3 (Supporting information Figure S1 in Appendix S1). The clustering patterns for *E. pinnatifida* were consistent with their geographical distributions (Figure 2). In the case of *E. stellata*, all the seven Russian populations (SR1, SR2, SR3, SR5, SR7, SR8, and SR9) and the two Chinese populations (SCW, SCP) were assigned to cluster 1, and the others were assigned to cluster 2 (Supporting information Figure S1 in Appendix S1). From this data, it is inferred that the Chinese populations SCW and SCP are significantly different from the other Chinese populations.

In the SAMOVA for each species, the number of population groups with the highest *F*_CT_ value (the proportion of genetic variation among groups) was chosen for its best grouping. In the case of *E. byunsanensis* and *E.  pungdoensis*, two groups were identified—(a) BJ and (b) BA, BG, BM, BS, BU, P—with the highest *F*_CT_ value of 0.98465. This grouping was inconsistent with the results of STRUCTURE analysis in which BJ was clustered with three other populations. Interestingly, the population P, which exists on Pungdo, a small Korean island in the Yellow Sea, was grouped with other five *E. byunsanensis* populations, even though it's classified as separate species. For *E. pinnatifida*, the highest *F*_CT_ value (0.88036) was for five groups—(a) PS5; (b) PS2; (c) PH5; (d) PH8, PH9; (e) PY. For *E. stellata*, the optimal number of groups with the highest *F*_CT_ (0.84619) was two—(a) SCP and (b) all the other 19 populations. It appears that this grouping does not reflect any distinct geographical patterns, meaning closer investigation is required to determine the factors which differentiated SCP population from other *E. stellata* populations, especially Chinese populations.

In the AMOVA, which used the SAMOVA groupings, for *E. byunsanensis* and *E. pungdoensis*, 98.47% of all the variation was partitioned among population groups, 1.52% among populations within groups, and 0.02% within populations (Table 2). For *E. pinnatifida*, 88.04% of the variation was distributed among population groups, compared to 84.62% for *E. stellata* (Table 2).

**Table 2:** Analysis of molecular variance (AMOVA) in four *Eranthis* species based on chloroplast microsatellites. The groups of the populations were defined according to SAMOVA results

	*df*	Sum of squares	Variance components	Percentage of variation
*Eranthis byunsanensis*, *Eranthis pungdoensis* (2 groups)				
Among groups	1	84055.962	1983.51034	98.47
Among populations within groups	5	4265.180	30.50015	1.51
Within populations	185	76.124	0.41148	0.02
Total	191	88397.266	2014.42198	100.00
*Eranthis stellata* (2 groups)				
Among groups	1	15047.027	264.56900	84.62
Among populations within groups	18	17555.038	33.58061	10.74
Within populations	552	8008.514	14.50818	4.64
Total	571	40610.579	312.65779	100.0
*Eranthis pinnatifida* (5 groups)				
Among groups	4	323.803	2.44326	88.04
Among populations within groups	1	0.483	0.00522	0.19
Within populations	165	53.924	0.32681	11.78
Total	170	378.211	2.77529	100.00

**Figure 4:**
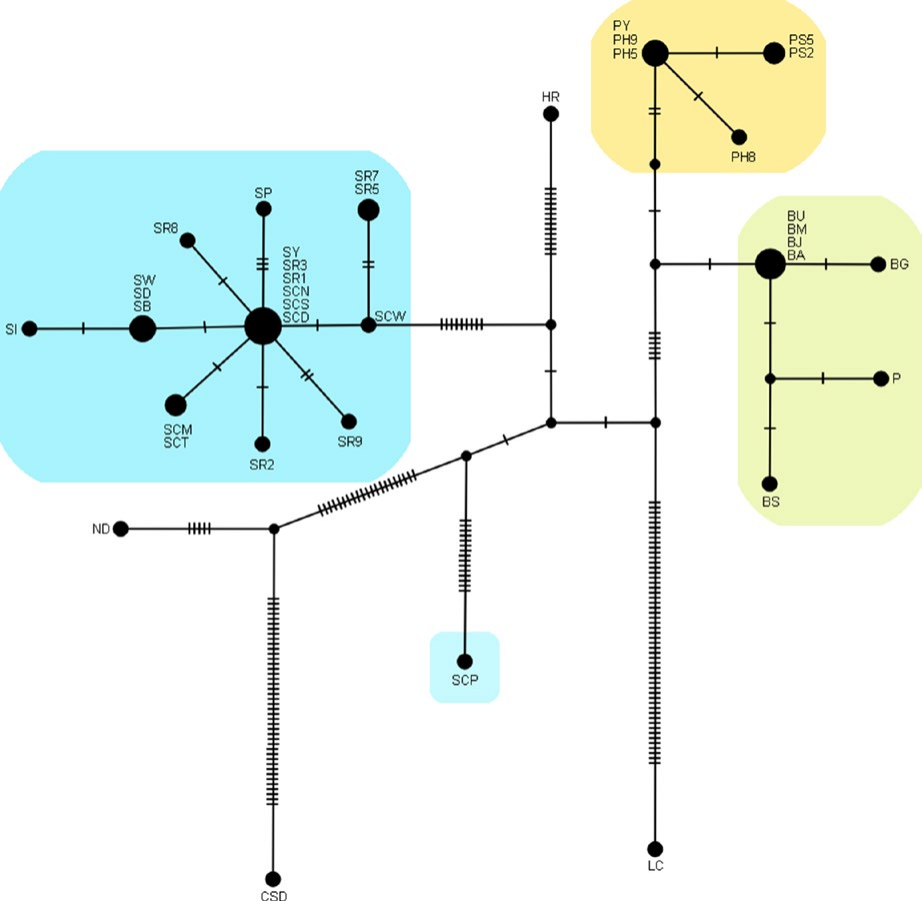
Median‐joining network of 37 sequences of concatenated chloroplast regions, one sequence from each population in the four *Eranthis* species. The size of the circle is proportional to the number of sequences which are considered as same by the POPART software used, and the population names for the sequences are shown beside the circles

In the median‐joining network which includes 35 sequences of six *Eranthis* species and one sequence each from *C. simplex* and *A. asiatica*, the sequences of *E. stellata*, *E. byunsanensis *(including *E. pungdoensis*), and *E. pinnatifida* were distinctly grouped by species (Figure 4), except for the Chinese population SCP, which was not grouped with other *E. stellata* populations (Figure 4). In this network, the population SCP behaved as if it was a separate species. The star‐like network in *E. stellata* indicates that this species has experienced population expansion (Figure 4).

### 3.3 Phylogenetic inferences and divergence time estimation

**Figure 5:**
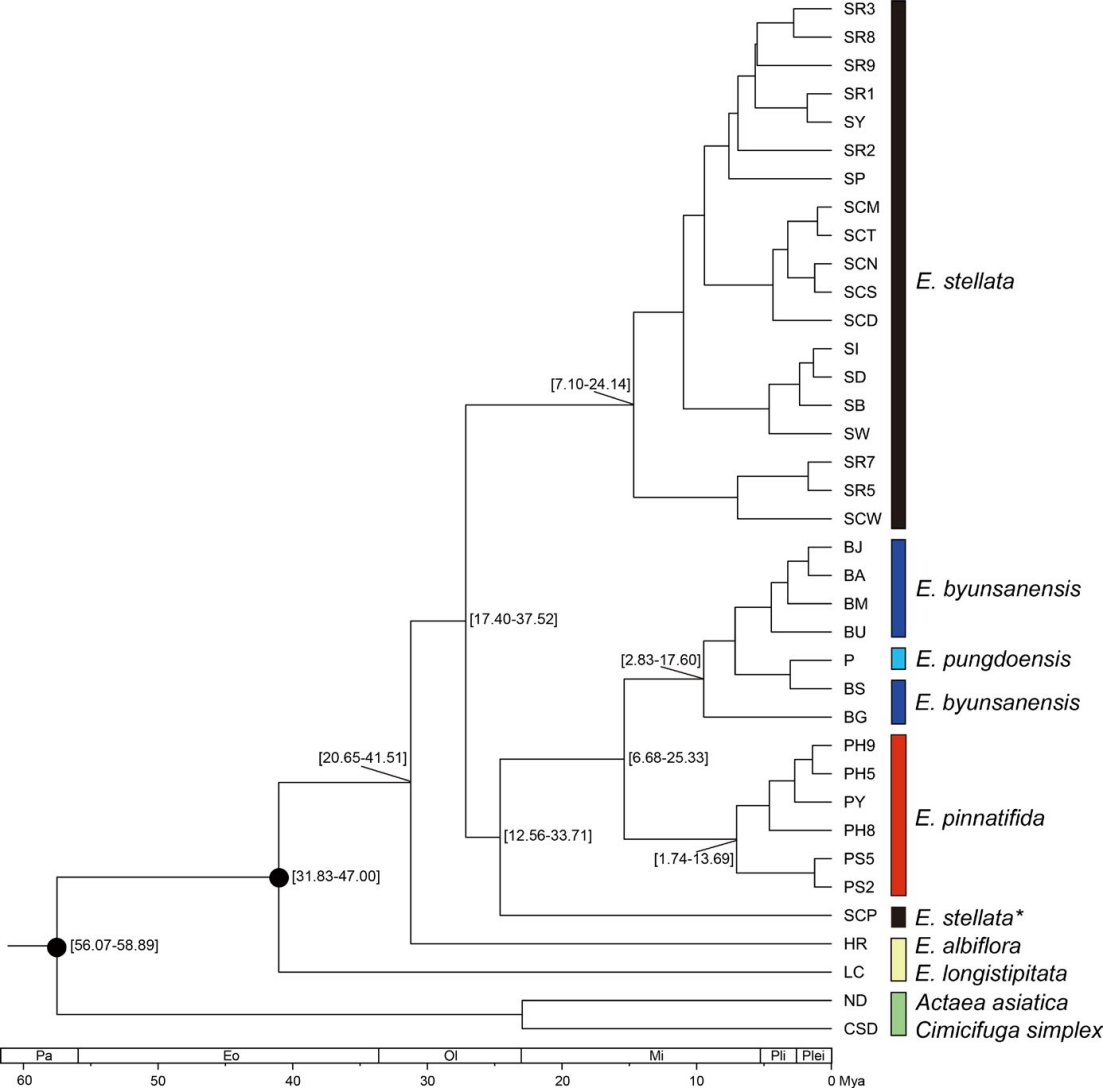
Phylogenetic relationships between the sequences (one sequence from each population) in the four *Eranthis* species constructed with BEAST. The numbers in brackets show 95% HPD of the divergence times, and two black circles on the nodes denote the calibration points. * mark beside *E. stellata* for SCP population indicates this population's different genetic characteristics from other *E. stellata* populations

The Bayesian analysis of phylogenetic relationships including 37 concatenated sequences of *rpl16* intron and *petL*‐*psbE* region showed that the *Eranthis* species are generally monophyletic, except for *E. stellata *(Figure 5), in which the Chinese population SCP was clustered with *E. byunsanensis* and *E. pinnatifida*, rather than with other *E. stellata* populations, and thus, is a paraphyletic group. *E. byunsanensis* and *E. pinnatifida* appear to be sister species, while *E. pungdoensis* was nested within the *E. byunsanensis* clade.

Maximum likelihood analyses in the software RAxML, which used *rpl16* intron region and *petL*‐*psbE* intergenic region separately, produced noticeably different tree topology to that from Bayesian inference using concatenated sequences (Figure 5; Supporting information Figure S2 and S3 in Appendix S1). This observation indicates that even though the two regions of *rpl16* intron and *petL*‐*psbE* intergenic spacer have a similar chloroplastic noncoding nature, their evolutionary patterns are not necessarily closely related. Despite this, we used almost all of the phylogenic reconstructions arising from our data analyses, unless there seemed to be extreme conflicts or contradictions. In these ML phylogenies, SCP population again behaved like another *Eranthis* species.

Molecular dating of the Bayesian phylogenetic tree using two secondary calibration points revealed that the divergences between *Eranthis* species or populations ranged from the Eocene to the Pleistocene (Figure 5). The split between *E. stellata* (with the exception of the SCP population) and the MRCA of *E. pinnatifida*, *E. byunsanensis*, and the population SCP, appears to have taken place during the early Oligocene (27.15 mya). The divergence between the population SCP and the clade including *E. byunsanensis*, *E. pinnatifida* is likely to have occurred during the late Oligocene (24.60 mya). The divergence of *E. pinnatifida* and *E. byunsanensis* dates to the early Miocene (15.38 mya), showing reciprocal monophyly. In each species of *Eranthis*, many splits between the populations took place during the Pleistocene. In the case of *E. stellata*, interestingly, clustering patterns of some populations from China, Korea, and Russia were quite inconsistent with their geographical distributions (Figure 5).

### 3.4 Ancestral area reconstruction

**Figure 6:**
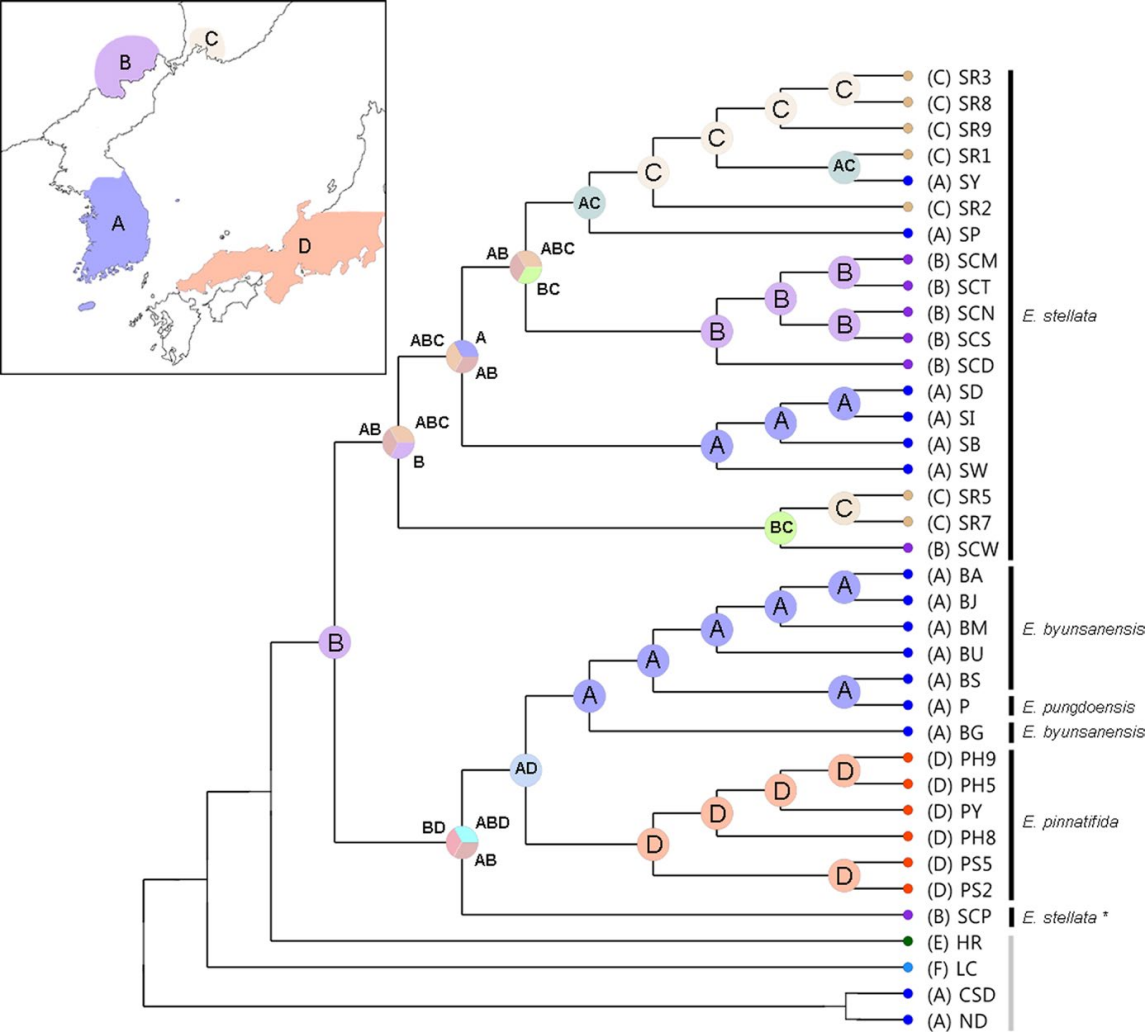
Ancestral area reconstructions performed with S‐DIVA in RASP. The map shows the four ranges (a‐d) defined from the sampling strategy in this study. Pie charts at the nodes of the tree show the possible ancestral ranges, for which in this analysis, the maximum number was set to four

S‐DIVA analysis revealed that the divergence between Korean endemic *Eranthis* species (A)—*E. byunsanensis*, *E. pungdoensis* ‐ and Japanese endemic *E. pinnatifida* (D) was a vicariant event, and that in *E. stellata*, some divergences between Chinese (B), Korean (A), and Russian (C) populations were also vicariances (Figure 6). For the four focal *Eranthis* species, Chinese range (B) was determined as an ancestral range. Overall, seven dispersal events and ten vicariant events are thought to have occurred.

## 4 DISCUSSION

### 4.1 The origin of *E. byunsanensis*

The theory that populations with a long evolutionary history show greater genetic diversity (Huang, Chiang, Schaal, Chou, & Chiang, 2001) suggests that the Jeju population, which exhibited the highest genetic diversity and is southernmost among all *E. byunsanensis* and *E. pungdoensis* populations, is the oldest of these populations. This means that the populations of these two species have primarily dispersed northward, even though it is fundamentally possible for them to disperse in any number of directions over a sufficiently long timescale. In contrast, according to our phylogeny reconstructions, the Jeju population diverged during Pleistocene (Figure 5), almost lastly among all the populations of *E. byunsanensis* and *E. pungdoensis *(Figure 5; Supporting information Figure S2 in Appendix S1), which is inconsistent with the abovementioned assumption regarding population age and genetic diversity. This observation of phylogeny can partially support southward dispersal of this species, although more evidence is needed for drawing conclusion.

The northward dispersal of *E. byunsanensis *enables the establishment of two different hypotheses regarding which species and region it speciated from. *E. byunsanensis* could have speciated from an *Eranthis* species in Japan, or one in China. The westward dispersal and following speciation from a Japanese species (assuming that it was the Japanese endemic *E. pinnatifda *and not an earlier common ancestor) can be refuted by the Bayesian phylogeny, in which these two species are sister taxa, and the crown group of *E. pinnatifida* (7.03 mya) is slightly younger than that of *E. byunsanensis* (9.47 mya) (Figure 5). In contrast, if *E. byunsanensis* speciated from Chinese *Eranthis* species, it's possible that this *Eranthis* species might have been distributed in the east of Sichuan (the habitat of current Chinese *E. albiflora*), in the western sea of the Korean peninsula, and in East China Sea, since at some points in the past, these regions were all connected by land bridge (Ota, 1998). However, since there is no previous study that there is, or has been *Eranthis* species on the east of Sichuan, western sea, and East China Sea, we cannot make a conclusion regarding the evolutionary history, dispersal and extinction of this “Chinese” *Eranthis* species.

The Jeju island, which exhibited high genetic diversity, and some regions nearby, might have been colonized by *Eranthis* species a long time ago, and may have acted as a dispersal route for this species and many other plants. In particular, *E. byunsanensis* inhabits many southern Korean islands between the Korean peninsula and the Jeju island, suggesting that in the past when Jeju and the Korean peninsula were connected, many populations of *E. byunsanensis* were located in the southern regions near the Jeju island. When assuming the speciation from an *Eranthis* species in eastern China, western sea of Korea, or East China Sea, even though we cannot conjecture the exact location of the speciation event, we can at least roughly infer that most probable site of speciation would have been near Jeju island. In the previous study, which analyzed genetic variation in *E. byunsanensis*, *E. pungdoensis,* and *E. pinnatifida*, (Lee et al., 2012) the TCS haplotype network revealed that Jeju was the likely center of differentiation for East Asian *Eranthis* species, and this conclusion is consistent with our results regarding the speciation of *E. byunsanensis*. In SAMOVA, Jeju population was differentiated from the other six populations, possibly due to its high genetic diversity. This result can also indicate that, after the geographical isolation of Jeju, genetic differentiation has accumulated, maybe due to genetic drift to some extent, over the period of less than 10,000 years between the sea level rising during the Holocene and the present day.

There is one more possibility regarding the speciation of *E. byunsanensis*—it may have speciated from *E. stellata,* which is found in Russia, China, North Korea, and South Korea. The distributional patterns of *E. stellata* and *E. byunsanensis* on the Korean peninsula seem to show allopatry, in which *E. stellata* distributes on mountain ranges with high altitudes, and *E. byunsanensis* on the regions with relatively low altitudes in South Korea (See Supporting information Figure S4 in Appendix S1). If these distributional patterns indicate allopatric speciation of *E. byunsanensis* from *E. stellata*, this speciation might have occurred after a Korean geological event, Tertiary tilted flexure (Shin & Hwang, 2014). In this event, the mountain ranges running in a north‐south direction were uplifted on the eastern side of the Korean peninsula, tilting the topography. This theory is concordant with current distributions of the two species. The tilted flexure of the Korean peninsula is inferred to have started about 23 mya in the early Miocene (Shin & Hwang, 2014). However, according to Bayesian phylogenetic analysis, the divergence between *E. stellata* and *E. byunsanensis* occurred prior to this time, at about 27.15 ± mya, in the Oligocene (Figure 5). As a possible explanation, *E. stellata* and *E. byunsanensis* might have diverged for some reasons prior to the tilted flexure, and then their distributions might have been affected by environmental divergence following the uplift event, presenting allopatric pattern. However, given that the factors which might have caused these two species’ divergence in the Oligocene is unknown, the speciation of *E. byunsanensis* from *E. stellata* may be considered unlikely. This is also supported by STRUCTURE analysis (Figure 3), in which the genetic structures for *E. stellata* and *E. byunsanensis* are significantly different from each other.

### 4.2 The origin of *E. pinnatifida*

For *E. pinnatifida*, which inhabits the central and southern parts of Honshu, there is high possibility that the populations dispersed northward after speciation. This is evidenced by Bayesian and maximum likelihood phylogenetic analyses, in which the northernmost two populations of Shiga prefecture (PS2 and PS5) were the last to diverge of the six *E. pinnatifida* populations (Figure 5; Supporting information Figure S2 and S3 in Appendix S1). This was observed in all the phylogenies constructed in this study. From the analysis of genetic diversity, it seems clear that the three Hiroshima populations (PH5, PH8, and PH9), which have more southern distributions than the Shiga populations, have a longer evolutionary history than the other three populations, weakly supporting this hypothesis as well. Also, the fact that this species does not inhabit northern Honshu and Hokkaido partially supports this northward dispersal hypothesis.

The northward dispersal of *E. pinnatifida* suggests that this species originated from other *Eranthis* species in the western regions of Japan, that is, near the Korean peninsula or China. *E. pinnatifida* might have originated from Korean endemic *E. byunsanensis*, or from *Eranthis* species in China. The first case is unlikely when considering that *E. byunsanensis* and *E. pinnatifida* are sister species. Moreover, the phylogeny indicates that *E. byunsanensis* and *E. pinnatifida* speciated at the similar time, although there is a clear time interval (Figure 5). Maybe, more probable explanation would be that *E. pinnatifida* originated from an *Eranthis* species in China or in the vicinity of China, and later speciated into its current form. Moreover, the petal morphology of *E. pinnatifida* and *E. albiflora* in Sichuan is rather similar, while that of *E. byunsanensis* is significantly different from these two species (Figure 2b,e,f). From this morphological perspective, it is not likely that *E. pinnatifida* evolved from *E. byunsanensis*, and it can be inferred that *E. byunsanensis* and *E. pinnatifida* both originated from a species on or near China, and sometime after, they speciated into divergent sister species. The result of S‐DIVA analysis suggests that both regions near South Korea and near Japan could have been colonized by common ancestor of these two species in the middle Miocene, about 15.38 mya (Figure 5). The factors which might have led to the speciation of *E. byunsanensis* and *E. pinnatifida* are difficult to identify from current restricted data; however, it can be inferred that the tendency of these two species to disperse northward and the restricted gene flow between these two species during the disconnection of Korea and Japan may have played a role to some extent. When comparing the distributional regions of *E. byunsanensis* and *E. pinnatifida*, the latitudinal ranges of these two species are quite similar, which suggests their niche similarity and the close relationships between these two species (their similar genetic structure is also indicated in STRUCTURE analysis (Figure 3)), though this should be accepted cautiously.

In addition, we investigated the possibility of southward dispersal of *E. pinnatifida*, even though the absence of this species from northern Honshu and Hokkaido can be interpreted in a number of ways, and cannot be currently explained. According to unpublished data, *E. stellata* can be found in Sakhalin; because Sakhalin, Hokkaido, and Honshu were connected at some points in the past, (Igarashi & Zharov, 2011; Ohnishi, Uno, Ishibashi, Tamate, & Oi, 2009; Suzuki et al., 2004) the speciation of *E. pinnatifida* from northern *E. stellata* in these regions cannot be completely dismissed. Moreover, the petal morphologies of *E. stellata* and *E. pinnatifida* are rather similar (Figure 2d,e), supporting to a minor degree the speciation from *E. stellata*. However, more decisive evidence for this hypothesis is needed for meaningful conclusion.

### 4.3 The origin of *E. stellata* and* E. pungdoensis*

When considering the wide distributional range of *E. stellata*, which covers Korea, China, and the eastern‐most regions of Russia, and the fact that the sampling of *E. stellata* is incomplete, it is difficult to determine from where and from what species *E. stellata* originated. However, according to our genetic diversity data, Russian populations generally exhibited the highest genetic diversity, indicating a long evolutionary history and raising the possibility of southward dispersal. The fact that the southern limit of *E. stellata* is in the South Korean mainland and that it is absent from southern islands, including Jeju, partially support its southward colonization. To obtain a clearer understanding of the origin and speciation of this species, the genetic structure and genetic diversity of other *Eranthis* species in Russia and China, namely *E. sibirica* and *E. albiflora*, need to be investigated. Other *E. stellata* populations in the western regions around the sea of Okhotsk and Sakhalin should be analyzed as well.

In this study, most of the analyses, including phylogenetic reconstructions, indicate that *E. pungdoensis* is genetically very similar to *E. byunsanensis* (Figure 5; Supporting information Figure S2 and S3 in Appendix S1), and is nested within *E. byunsanensis* in the phylogenies (Figure 5; Supporting information Figure S2 in Appendix S1). A previous study also reported a similarity between the two species using cpDNA sequences and ITS regions (Lee et al., 2012). This may clearly suggest *E. pungdoensis* speciated from *E. byunsanensis*.

Pungdo, the small western island in Korea, might have been connected to the Korean peninsula for a long time, during the Last Glacial Maximum (LGM; when the sea level was lower and the land bridge existed; Millien‐Parra & Jaeger, 1999), and long before LGM, considering its proximity to the Korean peninsula (Ota, 1998). In the Holocene, when sea level rose and Pungdo became isolated, the gene flow between Pungdo and the Korean peninsula was blocked, and this may have facilitated the allopatric speciation of *E. pungdoensis*. Morphologically, *E. pungdoensis* has larger petals than *E. byunsanensis*, however, there is no other clear difference between these two species (Oh & Ji, 2009; Figure 2a,b). This slight difference is consistent with the short period of genetic isolation.

Taxonomically, the results in this study also suggest that *E. pungdoensis* may be a variety of *E. byunsanensis*. The phylogeny in which the population of *E. pungdoensis* is nested within the populations of *E. byunsanensis* indicates that *E. pungdoensis* may belong to *E. byunsanensis*. When considering their morphological differences, including the differences in their petal shape and size, reasonable taxonomic treatment for *E. pungdoensis* may require further observations of other morphological, genetic features which have not been investigated yet.

### 4.4 The genetic characteristics of the Chinese population SCP

According to the results of STRUCTURE analysis, SAMOVA, network analysis, and phylogenetic inferences, the Chinese population SCP is markedly different from other Chinese *E. stellata* populations and all other sampled *E. stellata* populations. In these analyses, SCP population was included in the genus *Eranthis*, but clearly does not belong in *E. stellata*.

The reason for the extremely large difference in SCP population is not easy to infer. To ensure that there was no error in the sampling process, we extracted genomic DNA again from other bract samples from the SCP population, while also closely examining the morphology of the bracts. However, this follow‐up analysis yielded the same results.

In the Bayesian phylogeny, the SCP population diverged from the clade of *E. byunsanensis* and *E. pinnatifida* during the late Oligocene (24.60 mya), this divergence time not being very far from the divergence between other *E. stellata* populations and the clade of *E. byunsanensis*, *E. pinnatifida*, and SCP population (Figure 5). In the maximum likelihood phylogeny, the SCP population diverged from the four *Eranthis* species a long time ago, close to the time when *E. albiflora* diverged from those four *Eranthis* species (Supporting information Figures S2 and S3 Appendix S1). From these observations, we suggest that the SCP population has the genetic characteristics of an ancient *Eranthis* species, even though we cannot clearly conclude which *Eranthis* species this may have been. According to the phylogenies, the SCP population may be genetically rather similar to *E. albiflora*, considering their close divergence times. S‐DIVA analysis also supports this argument. The results of S‐DIVA analysis suggest that the ancestral location for the common ancestor of the four *Eranthis* species may have been in China (Figure 6). This provides evidence for the possibility that Chinese *E. stellata* is the most ancestral population among all these four *Eranthis* species. Even though the highest genetic diversity was found in Russian *E. stellata* populations, and the sampling of *E. stellata* in Russia and North Korea is incomplete, this argument should be seriously considered.

Given the unique genetic characteristics of the SCP population, if these characteristics represent ancestral genetic traits, then we can suggest that it is an extremely stable population that has undergone a very slow genetic evolution. An alternative hypothesis is that the SCP population has genetically evolved rapidly for some reasons, resulting in the high level of differentiation, even though there are no geological or ecological factors which may have driven this. Another interpretation, considering that the bract and flower morphology of the SCP population is the same as the other *E. stellata* populations, is that the SCP population may be a cryptic species of *E. stellata*, even though explaining this is challenging—the explanation would have to involve a very rare mutational event.

Again, the genetic characteristics of the SCP population should be interpreted with caution, partly because these results depend on only chloroplast DNA, even though it is meaningful that chloroplast noncoding sequences and chloroplast microsatellites presented the concordant results. Related experiments using nuclear and mitochondrial DNA should be conducted for clear identification of SCP population and definite conclusions on its molecular evolution.

## 5 CONCLUSION

When combining the possible origins of these *Eranthis* species described above, it can be concluded that the Korean peninsula and nearby regions harbor northern‐sourced (*E. stellata*) and southern‐sourced (*E. byunsanensis*, *E. pungdoensis, E. pinnatifida*) species of *Eranthis*, and these regions are phylogeographically valuable in terms of determining complex origin patterns for closely related species. Also, the observation of unique Chinese SCP population provides the opportunity to infer the ancestral location for these four focal species, thereby adding an important description to their evolutionary history.

## CONFLICT OF INTEREST

None declared.

## AUTHOR CONTRIBUTION

Byoung‐Un Oh contributed to project design, sample collection and writing manuscript. Ami Oh contributed to project design, sample collection, lab work, data analysis and writing manuscript.

## Data Availability

DNA sequences:GenBank accessions: MH660625‐MH660701.
