# Supplementary material for: The speciation history of northern‐ and southern‐sourced Eranthis (Ranunculaceae) species on the Korean peninsula and surrounding areas
Source: Ecol Evol. 2019 Feb 14;9(5):2907–19. doi: 10.1002/ece3.4969 (PMC6405485; doi:10.1002/ece3.4969)
Supplement: Supplementary file 1 [file ECE3-9-2907-s001.docx]

*Ecology and Evolution*

**SUPPORTING INFORMATION**

**The speciation history of northern- and southern-sourced *Eranthis* species on the Korean peninsula and surrounding areas**

Ami Oh, Byoung-Un Oh

**APPENDIX S1**

**Table S1** Information on the populations of *Eranthis* species used in this study.

| **Species** | **Sampling locations** | **Geographical coordinates** | **altitude** | **Code** |
| --- | --- | --- | --- | --- |
| *E. byunsanensis* | Jinan, Korea | N 35˚45´38.0˝ E 127˚24´51.7˝ | 503m | BM |
|  | Ulsan, Korea | N 35˚34´39.2˝ E 129˚25´24.1˝ | 96m | BU |
|  | Gapyeong, Korea | N 37˚55´04.7˝ E 127˚25´07.1˝ | 753m | BA |
|  | Jeju, Korea | N 33˚26´10.4˝ E 126˚37´42.4˝ | 576m | BJ |
|  | Sokcho, Korea | N 38˚10´29.8˝ E 128˚29´07.4˝ | 225m | BS |
|  | Yesan, Korea | N 36˚42´41.3˝ E 126˚37´02.6˝ | 240m | BG |
| *E. pungdoensis* | Pungdo Is, Ansan, Korea | N 37˚06´36.9˝ E 126˚23´11.9˝ | 127m | p |
| *E. stellata* | Wonju, Korea | N 37˚15´36.6˝ E127˚57´51.0˝ | 682m | SW |
|  | Inje, Korea | N 38˚02´35.3˝ E128˚29´44.6˝ | 944m | SI |
|  | Danyang, Korea | N 36˚59´29.2˝ E128˚15´56.4˝ | 572m | SD |
|  | Namyangju, Korea | N 37˚41´16.2˝ E 127˚15´09.1˝ | 244m | SP |
|  | Yeongdong, Korea | N 36˚02´00.9˝ E 127˚50´24.8˝ | 881m | SY |
|  | Yeongcheon, Korea | N 36˚10´17.3˝ E 128˚59´41.8˝ | 758m | SB |
|  | Wangqing, Jilin, China | N 43˚18´15.4˝ E 129˚18´53.4˝ | 474m | SCW |
|  | Antu, Jilin, China - 1 | N 42˚58´48.3˝ E 128˚41´22.4˝ | 553m | SCM |
|  | Antu, Jilin, China - 2 | N 42˚36´29.1˝ E 128˚00´52.6˝ | 515m | SCS |
|  | Antu, Jilin, China - 3 | N 42˚17´40.8˝ E 127˚49´15.9˝ | 1096m | SCT |
|  | Fusong, Jilin, China | N 41˚51´47.5˝ E 127˚41´51.3˝ | 960m | SCN |
|  | Antu, Jilin, China - 4 | N 42˚30´39.9˝ E 128˚30´58.5˝ | 681m | SCD |
|  | Helong, Jilin, China | N 42˚35´37.4˝ E 128˚54´20.5˝ | 631m | SCP |
|  | Nadezhdinsky, Primorskiy kray, Russia | N 43˚34´17.8˝ E 131˚51´10.6˝ | 5m | SR1 |
|  | Khasanskiy, Primorskiy kray, Russia | N 43˚21´28.9˝ E 131˚39´15.7˝ | 39m | SR2 |
|  | Vladivostok, Primorskiy kray, Russia | N 43˚12´42.4˝ E 132˚04´12.5˝ | 81m | SR3 |
|  | Shkotovskiy, Primorskiy kray, Russia - 1 | N 43˚32´21.8˝ E 132˚25´07.4˝ | 129m | SR5 |
|  | Shkotovskiy, Primorskiy kray, Russia - 2 | N 43˚35´21.7˝ E 132˚27´55.4˝ | 162m | SR7 |
|  | Partizansk, Primorskiy kray, Russia | N 43˚20´34.1˝ E 132˚57´24.4˝ | 514m | SR8 |
|  | Gorod Artem, Primorskiy kray, Russia | N 43˚16´30.1˝ E 132˚13´46.3˝ | 45m | SR9 |
| *E. pinnatifida* | Inukami, Shiga Pref., Japan | N 35°14'34.3" E 136°19'31.7" | 345m | PS2 |
|  | Higashi-Ohmi-shi, Shiga Pref., Japan | N 35°02'39.9" E 136°19'26.4" | 319m | PS5 |
|  | Syobara-shi, Hiroshima Pref., Japan | N 34°46'10.3" E 133°06'20.6" | 317m | PH5 |
|  | Miyoshi-shi, Hiroshima Pref., Japan - 1 | N 34°45'48.9" E 132°59'48.8" | 265m | PH8 |
|  | Miyoshi-shi, Hiroshima Pref., Japan - 2 | N 34°45'05.6" E 132°47'49.6" | 176m | PH9 |
|  | Iwakuni-shi, Yamaguchi Pref., Japan | N 34°15'19.6" E 131°57'19.0" | 138m | PY |
| *E. albiflora* | Hailuogou, Sichuan, China | N 29˚35´02.2˝ E 102˚01´27.3˝ | 2761m | HR |
| *E. longistipitata* | Bostanlik, Uzbekistan | N 41˚31´24.1˝ E 70˚01´11.3˝ | 1678m | LC |
| *E. hyemalis* | Saint-Paul-de-Monestier, France | N 44°54'42.1" E 5°37'11.5˝ | 928m | HYE |

**Figure S1** Histograms of genetic assignment analyses with STRUCTURE for each *Eranthis* species, *E. stellata*, *E. byunsanensis*, *E. pungdoensis*, and *E. pinnatifida*. The population names are listed below the histograms.


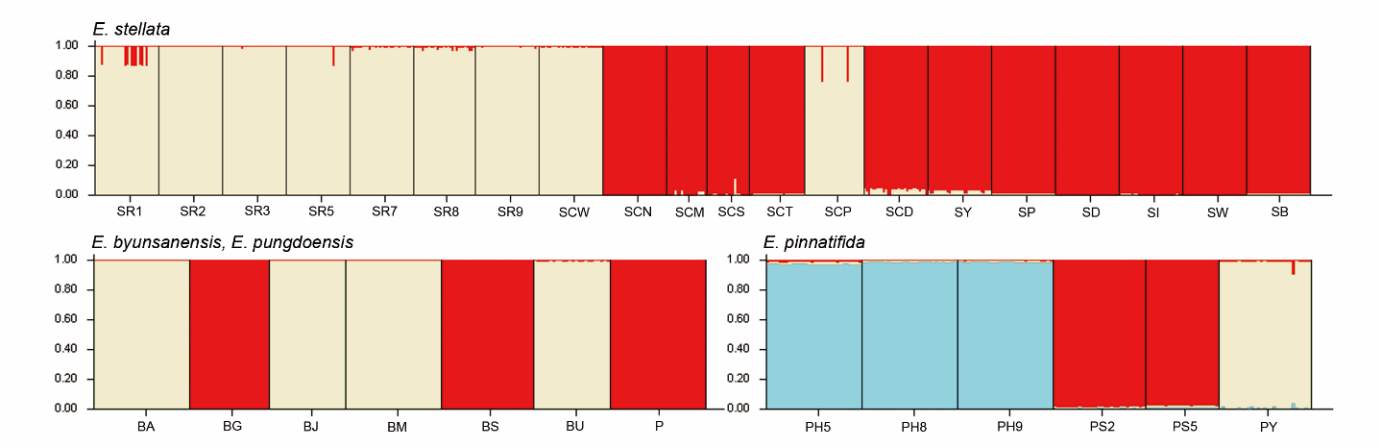


**Figure S2** Maximum likelihood phylogeny using *petL*-*psbE* intergenic regions.


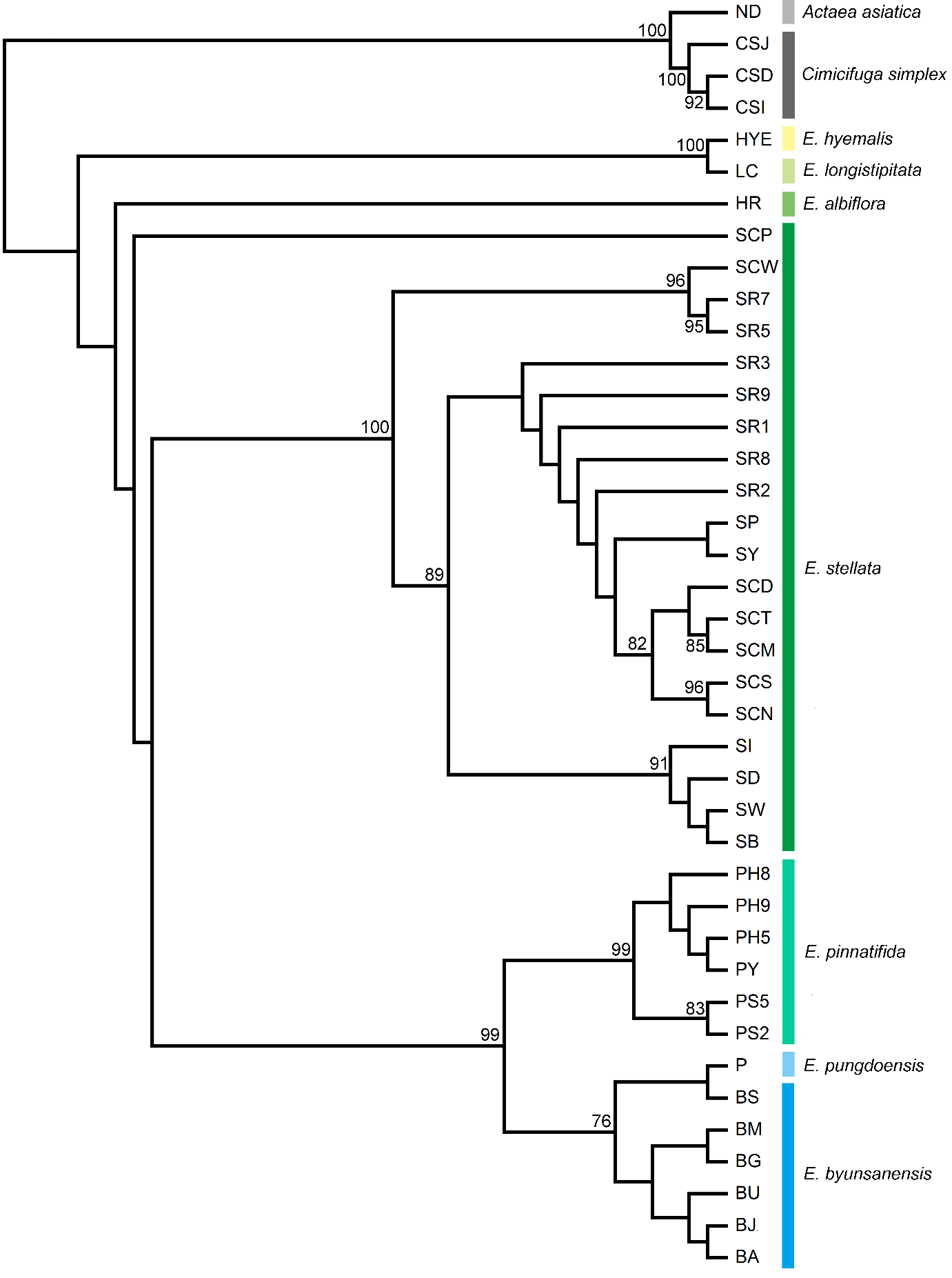


**Figure S3** Maximum likelihood phylogeny using *rpl16* introns.


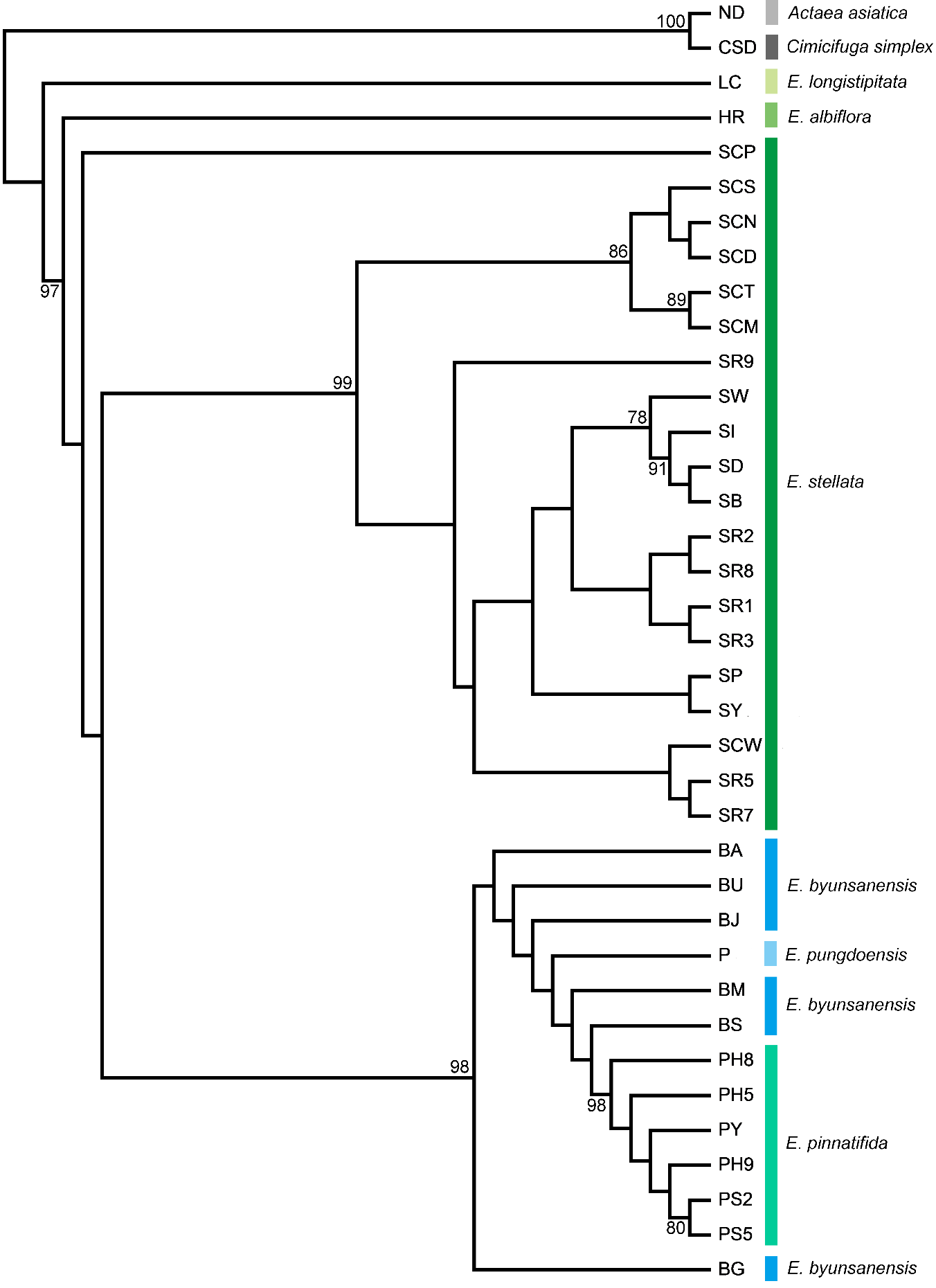


**Figure S4** The distribution maps for *Eranthis byunsanensis* and *E. stellata* in South Korea. The red dots denote the distribution localities of *E. byunsanensis*, and the black dots, *E. stellata* (modified from Oh et al., 2016).


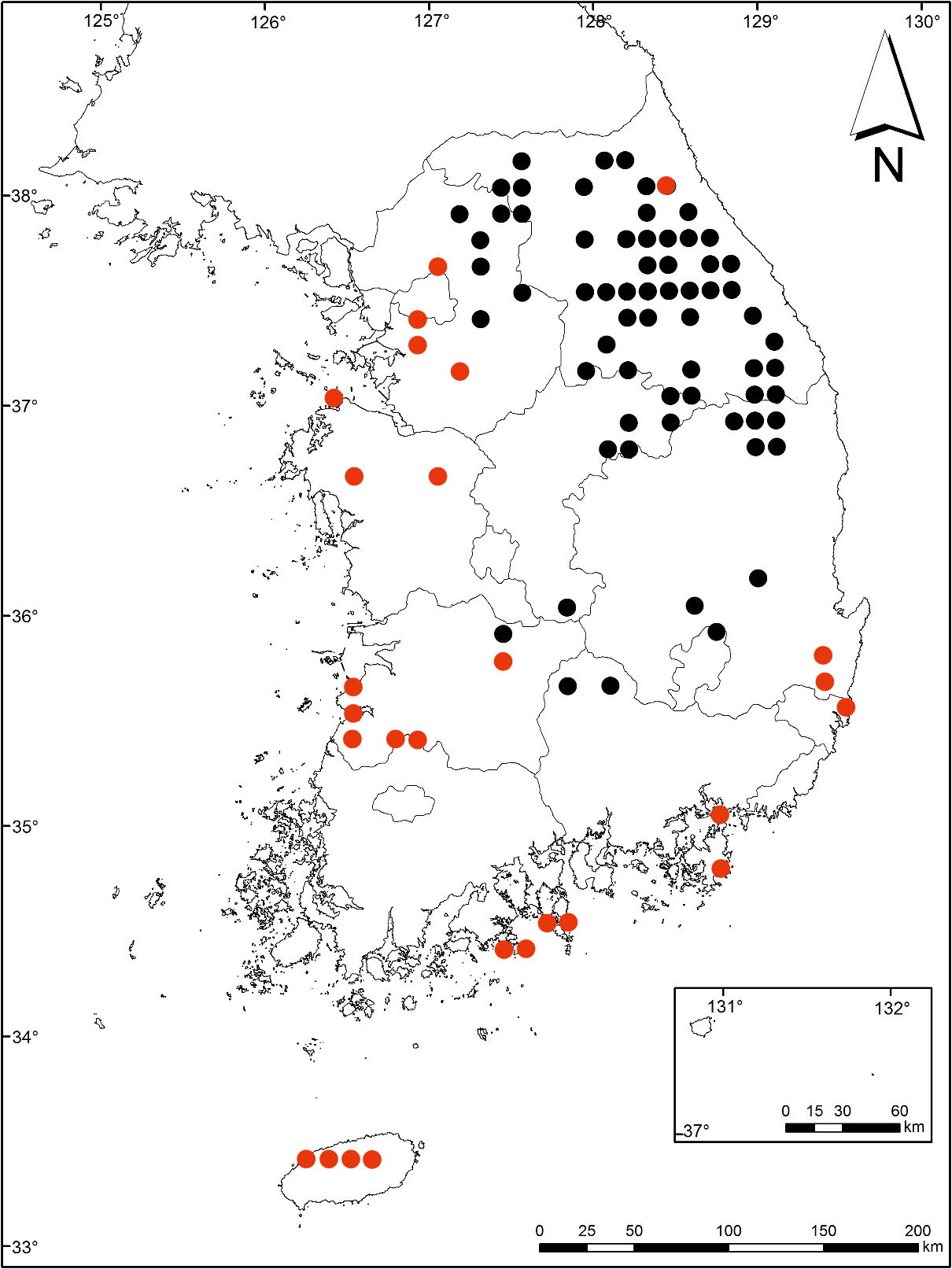


**Reference**

Oh, B. U., Ko, S. C., Kang, S. H., Paek, W. G., Yoo, G. O., Im, H. T., … Cho, D. G. (2016). Distribution maps of vascular plants in Korea. Korea National Arboretum, Pocheon, Korea.
